# Evaluation of the Degree of Agreement of Four Methods for Diagnosing Diabetic Autonomic Neuropathy

**DOI:** 10.3389/fneur.2021.637099

**Published:** 2021-05-25

**Authors:** Yu Peng, Ying-shan Liu, Min-yi Wu, Chu-na Chen, Chu-qiao Li, An-qi Jiang, Chong-xin Li, Yao Wang, Ge Tian, Su-yue Pan, Ling-ling Xu

**Affiliations:** ^1^Department of Neurology, Nanfang Hospital, Southern Medical University, Guangzhou, China; ^2^Department of Endocrinology, Shenzhen Hospital, Southern Medical University, Guangzhou, China; ^3^The Third School of Clinical Medicine, Southern Medical University, Guangzhou, China; ^4^Department of Cardiology, Nanfang Hospital, Southern Medical University, Guangzhou, China

**Keywords:** diabetic autonomic neuropathy, diagnostic methods, diagnosis agreement, COMPASS-31, Ewing's tests, heart rate variability, skin sympathetic reaction

## Abstract

**Background:** There are many methods to diagnose diabetic autonomic neuropathy (DAN); however, often, the various methods do not provide consistent results. Even the two methods recommended by the American Diabetes Association (ADA) guidelines, Ewing's test and heart rate variability (HRV), sometimes give conflicting results. The purpose of this study was to evaluate the degree of agreement of the results of the Composite Autonomic Symptom Score 31 (COMPASS-31), skin sympathetic reaction (SSR) test, Ewing's test, and HRV in diagnosing DAN.

**Methods:** Patients with type 2 diabetes were recruited and each received the COMPASS-31, SSR, Ewing's test, and HRV for the diagnosis of DAN. Patients were categorized as DAN(+) and DAN(–) by each of the tests. Kappa consistency tests were used to evaluate the agreement of diagnosing DAN between any two methods. Spearman's correlation test was used to evaluate the correlations of the severity of DAN between any two methods. Receiver operating characteristic (ROC) analyses were used to evaluate the diagnostic value and the cutoff value of each method.

**Results:** A total of 126 type 2 diabetic patients were included in the study. The percentages of DAN(+) results by HRV, Ewing's test, COMPASS-31, and SSR were 61, 40, 35, and 33%, respectively. COMPASS-31 and Ewing's test had the best agreement for diagnosing DAN (κ = 0.512, *p* < 0.001). COMPASS-31 and Ewing's test also had the best correlation with respect to the severity of DAN (*r* = 0.587, *p* < 0.001). Ewing's test and COMPASS-31 had relatively good diagnostic values (AUC = 0.703 and 0.630, respectively) in the ROC analyses.

**Conclusions:** COMPASS-31 and Ewing's test exhibit good diagnostic consistency and severity correlation for the diagnosis of DAN. Either test is suitable for the diagnosis of DAN and treatment follow-up.

## Introduction

Diabetic autonomic neuropathy (DAN) is one of the most common, chronic complications of diabetes mellitus (DM) (1), and DM is also the most common cause of chronic automatic neuropathy (2). Patients with DAN may present with dry skin with poor nutrition, persistent scarring, diarrhea/constipation, erectile dysfunction, resting tachycardia/bradycardia, orthostatic hypotension, painless myocardial ischemia, myocardial infarction, malignant arrhythmia, and even sudden cardiac death (3). The reported prevalence of DAN in diabetic patients is 17–73% (4–7), with the wide range attributable to factors such as different diagnostic criteria, age, and race. Since 2012, Ewing's test and heart rate variability (HRV) have been recommended by the American Diabetes Association (ADA) and the European Association for the Study of Diabetes (EASD) for diagnosing diabetic cardiac autonomic neuropathy (DCAN) (8, 9). However, in clinical practice, we have found that these two methods frequently provide different results in the same patient. In addition, Ewing's test and HRV are time-consuming and require advanced equipment to perform, which makes them relatively difficult to perform in practice.

The Composite Autonomic Symptom Score 31 (COMPASS-31) is a self-assessment instrument published by the Mayo Clinic in 2012 and includes 31 items assessing six domains of autonomic function (10). It is more convenient than its predecessors, the Autonomic Symptom profile composed of 169 items (ASP 169) and the COMPASS-72, and it has been proven to be suitable for the assessment of DAN or other small fiber polyneuropathies (SFPNs) (11, 12). Skin sympathetic reaction (SSR) is also a common and simple method to evaluate the function of sympathetic nerves and is a useful electrophysiological test for the early diagnosis of diabetic neuropathy (13). However, it is unclear whether COMPASS-31 or SSR is consistent with Ewing's test or HRV in diagnosing DAN.

Thus, the purpose of this study was to evaluate the degree of agreement of COMPASS-31, SSR, Ewing's test, and HRV in diagnosing DAN.

## Materials and Methods

### Subjects

Patients with type 2 DM were recruited from the Department of Endocrinology, Nanfang Hospital, between September 2017 and August 2018. The inclusion criteria for this study were: DM diagnosed based on the 1999 World Health Organization (WHO) criteria (14) and 18–80 years old. The exclusion criteria were: (1) peripheral neuropathy; (2) history of stroke; (3) history of heart disease; (4) loss of any extremities; and (5) unable to stand without assistance. Patient information collected included age sex, course of DM, family history, smoking history, drink history, medication, and body mass index (BMI). Laboratory testing included measurement of hemoglobin A1c (HbAlc) level and low-density lipoprotein (LDL) level. All patients received the four tests being evaluated: COMPASS-31, SSR, Ewing's test, and HRV. Patients were categorized as DAN(+) or DAN(–) based on the individual test results, as described below.

This study was approved by the Ethics Committee of Nanfang hospital (NFEC-2018-115), and all participants provided written informed consent.

### Composite Autonomic Symptom Score 31

As there was not a formal Chinese version and norm of the COMPASS-31, we recruited 84 healthy volunteers as a control group. We scored the COMPASS-31 results for each patient and healthy control subject by translating the English version into Chinese and inquiring every examinee rather than reading the scale by examinees. The original score for each domain and the weighted total score were recorded (10). The upper 95% confidence interval (CI) of the weighted total score of the control group was defined as the cutoff value for diagnosing DAN, and patients with a total score above the cutoff value were considered DAN(+).

### Skin Sympathetic Reaction

SSR was performed following the standard procedure described in the literature (13). The latencies and amplitudes of initiation were recorded with an electromyography machine (Dantec Keypoint 9033A, Copenhagen, Denmark). Abnormalities were defined by reference values established by the Peking Union Medical College Hospital for healthy Chinese people. An upper extremity latency >1,512 ms or an amplitude <484 μV was considered abnormal; a lower extremity latency >2,230 ms or an amplitude <364 μV was considered abnormal. The number of abnormal extremities was the total SSR score, which ranged from 0 to 4. Patients with a score ≥1 were considered DAN(+).

### Ewing's Test

Ewing's test has been used to evaluate the autonomic function of diabetic patients since the 1980s (15, 16). It consists of five tests. Three are predominantly parasympathetic tests: mean max/min ratio during three Valsalva maneuvers; mean max/min heart rate (HR) difference during six deep breaths; and the 30:15 ratio after standing. Two are predominantly sympathetic tests: the systolic blood pressure (BP) decrease after standing and the diastolic BP increase during a sustained handgrip. As the handgrip test is difficult to perform, only the other four tests are usually performed in clinical practice. The four tests (excluding the handgrip test) were performed using an electromyography machine (Dantec Keypoint 9033A, Copenhagen, Denmark) and a non-invasive BP monitoring system (Task Force Monitor, Finometer PRO, Netherlands). All patients were asked to refrain from caffeine, to not take β-blocker or angiotensin-converting enzyme inhibitor (ACEI) medications on the day of the testing, and to eat only a light breakfast. The tests were performed between 9:00 a.m. and 11:00 a.m. in a warm, quiet room. There was a 2-min rest period after each individual test. The results for each test were classified as normal, borderline, and abnormal and scored as 0, 0.5, and 1, respectively (Table 1). Thus, the total Ewing's score ranged from 0 to 4. Patients with a score ≥2 were classified as DAN(+) based on the ADA guidelines (8).

**Table 1:** The scored standards of each item for Ewing's test and HRV.

	**Normal (scored as 0)**	**Borderline (scored as 0.5)**	**Abnormal (scored as 1)**
**Ewing's test**			
Valsalva ratio	≥1.21	1.11–1.20	≤1.10
Deep breath (min^−1^)	≥15	11–14	≤10
30:15 ratio after standing	≥1.04	1.01–1.03	≤1.00
BP fall after standing (mmHg)	≤10	11–29	≥30
**HRV**			
SDNN (ms)	≥50	–	<50
SDANN (ms)	≥40	–	<40
RMSSD (ms)	≥15	–	<15
pNN50	≥0.75	–	<0.75
LF (ms^2^)	≥300	–	<300
HF (ms^2^)	≥200	–	<200

*HRV, heart rate variability; SDNN, standard deviation of the normal-to-normal interval; SDANN, standard deviation of the average NN interval; RMSSD, square root of the mean squared differences of successive NN intervals; pNN50, proportion of successive NN intervals >50 ms; LF, low-frequency power; HF, high-frequency power*.

### Heart Rate Variability

HRV has been used to evaluate the autonomic function of diabetic patients for many years (14). The standard deviation of the normal-to-normal (NN) interval (SDNN), the standard deviation of the average NN interval (SDANN), the square root of the mean squared differences of successive NN intervals (RMSSD), the proportion derived by dividing the number of interval differences of successive NN intervals >50 ms by the total number of NN intervals (pNN50) in the time-domain analysis, and the low-frequency (LF) and high-frequency (HF) power in the frequency-domain analysis are recommended as indicators for the diagnosis of DAN by the ADA (8). The sequence of the NN intervals in an entire 24-h period was recorded after the Ewing's test for all patients using a Holter recorder (Diagnostic Monitoring Software 300-4AL, Nevada, USA). The six recommended items were classified as normal and abnormal and scored as 0 and 1, respectively (Table 1). The sum of the six scores is the total HRV score, which ranges from 0 to 6. Patients with a total HRV score ≥2 were classified as DAN(+), as recommended by the ADA (8).

### Statistical Analysis

All statistical analyses were performed by using SPSS version 20.0 software (IBM Corp., Armonk, NY, USA). Data were expressed as the mean ± standard deviation or the median and interquartile range (IQR). Independent-samples non-parametric tests were performed to compare the results of diabetic patients and controls. Kappa consistency tests were performed to evaluate the consistency of diagnosing DAN between any two methods. The correlation of DAN severity between the different methods was evaluated with Spearman's correlation test. Receiver operating characteristic (ROC) analyses were used to evaluate the diagnostic value and the cutoff value of each method. A value of *p* < 0.05 was considered statistically significant.

## Results

A total of 126 patients with type 2 DM were included in the study. The demographic data, physical and biochemical characteristics, and autonomic nervous function evaluation of the DM group and the control group are summarized in Table 2. The cutoff value of the COMPASS-31 for diagnosing DAN in this study was 21.4, which was calculated from the control group. The numbers of DAN(+) patients diagnosed by HRV, Ewing's test, COMPASS-31, and SSR were 77 (61%), 51 (40%), 44 (35%), and 41 (33%), respectively. This result suggested that HRV had a higher diagnostic rate than the other three methods.

**Table 2:** The baseline characteristics of the diabetic patient group and healthy controls.

	**Total DM**	**DM divided by COMPASS-31**		**Healthy control**
		**DAN(+)**	**DAN(–)**	
*n*	126	44	82	84
Age (years)	59.5 ± 11.3	62.0 ± 11.0	58.2 ± 11.2^*^	40.5 ± 15.3^*^
Male/female	74/52	27/17	47/35	32/52
Course of disease (years)	8.5 (3.0–14.5)	8.5 (4.0–16.5)	8.5 (3.0–12.0)	–
Family history	21 (16.7%)	5(11.4%)	16 (19.5%)	–
Smoking history	44 (34.9%)	11(25.0%)	33(40.2%)	–
Drinking history	11 (8.7%)	2(4.5%)	9 (11.0%)	–
Hypertension history	67 (53.2%)	26 (59.1%)	41 (50.0%)	–
ACEI, ARB, or β-blocker use	24 (19.0)	9 (20.5%)	15 (18.3%)	–
BMI (kg/m^2^)	23.5 ± 3.2	23.7 ± 3.7	23.5 ± 3.0	–
HbAlc (%)	8.9 (7.2–10.6)	8.7 (7.2–10.4)	8.9 (7.2–10.7)	–
LDL (mmol/L)	2.9 (2.3–3.5)	2.6 (2.2–3.3)	3.0 (2.4–3.6)	–
Total COMPASS-31 score	15.8 (8.9–26.2)	32.3 (25.3–41.8)	11.3 (7.7–15.4)^‡^	7.3 (2.5–13.7)^‡^
Orthostatic intolerance	2 (0–4)	4 (4–5)	0 (0–2)^‡^	0 (0–2)^‡^
Vasomotor	0 (0–0)	0 (0–0)	0 (0–0)^†^	0 (0–2)^†^
Secretomotor	2 (1–3)	3 (2–5)	1 (1–2)^‡^	1 (0–1)^‡^
Gastrointestinal	3 (1–6)	6 (3–12)	2 (1–4)^‡^	2 (0–3)^‡^
Bladder	2 (0–2)	2 (1.5–2)	1 (0–2)^‡^	0 (0–0)^‡^
Pupillomotor	1 (0–2)	1 (0–3)	1 (0–1)^*^	1 (0–1)
HRV score	2 (1–2)	2 (1–2)	2 (1–2)	–
SDNN (ms)	107 (87–132)	105 (88–130)	108 (90–136)	–
SDANN (ms)	92 (70–112)	86 (62–105)	96 (72–113)	–
RMSSD (ms)	39 (25–62)	46 (31–72)	32 (23–53)^*^	–
pNN50	5 (1–10)	5 (3–13)	4 (1–10)	–
LF (ms^2^)	192 (102–334)	165 (57–287)	216 (122–336)	–
HF (ms^2^)	88 (49–151)	93 (35–146)	88 (54–153)	–
Ewing test score	1.5 (0.5–2.5)	3.0 (1.75–3.0)	1.0 (0.5–1.5)^‡^	–
SSR score	0 (0–2)	1 (0–2)	0 (0–0)^*^	–

*The health control group was compared with the total diabetic patients group. Patients were divided into a DAN(+) and a DAN(–) group by COMPASS-31. The DAN(+) subgroup was compared with the DAN(–) subgroup*.

*SDNN, standard deviation of the normal-to-normal interval; SDANN, standard deviation of the average NN interval; RMSSD, square root of the mean squared differences of successive NN intervals; pNN50, proportion of successive NN intervals >50 ms; LF, low-frequency power; HF, high-frequency power*.

* *p < 0.05*,

† *p < 0.01*,

‡ *p < 0.001*.

Based on the COMPASS-31 results, the patients were divided into DAN(+) and DAN(–), and their characteristics are compared in Table 2. Sex proportion, course of DM, family history, smoking, drinking, medication, BMI, and the HbAlc and LDL levels were not different between the two groups (all, *p* > 0.05). However, the mean age of DAN(+) patients was greater than that of DAN(–) patients (*p* < 0.05). With respect to autonomic nervous function evaluation, DAN(+) patients had significantly higher Ewing's test scores and SSR scores than DAN(–) patients, but not HRV scores.

**Figure 1:**
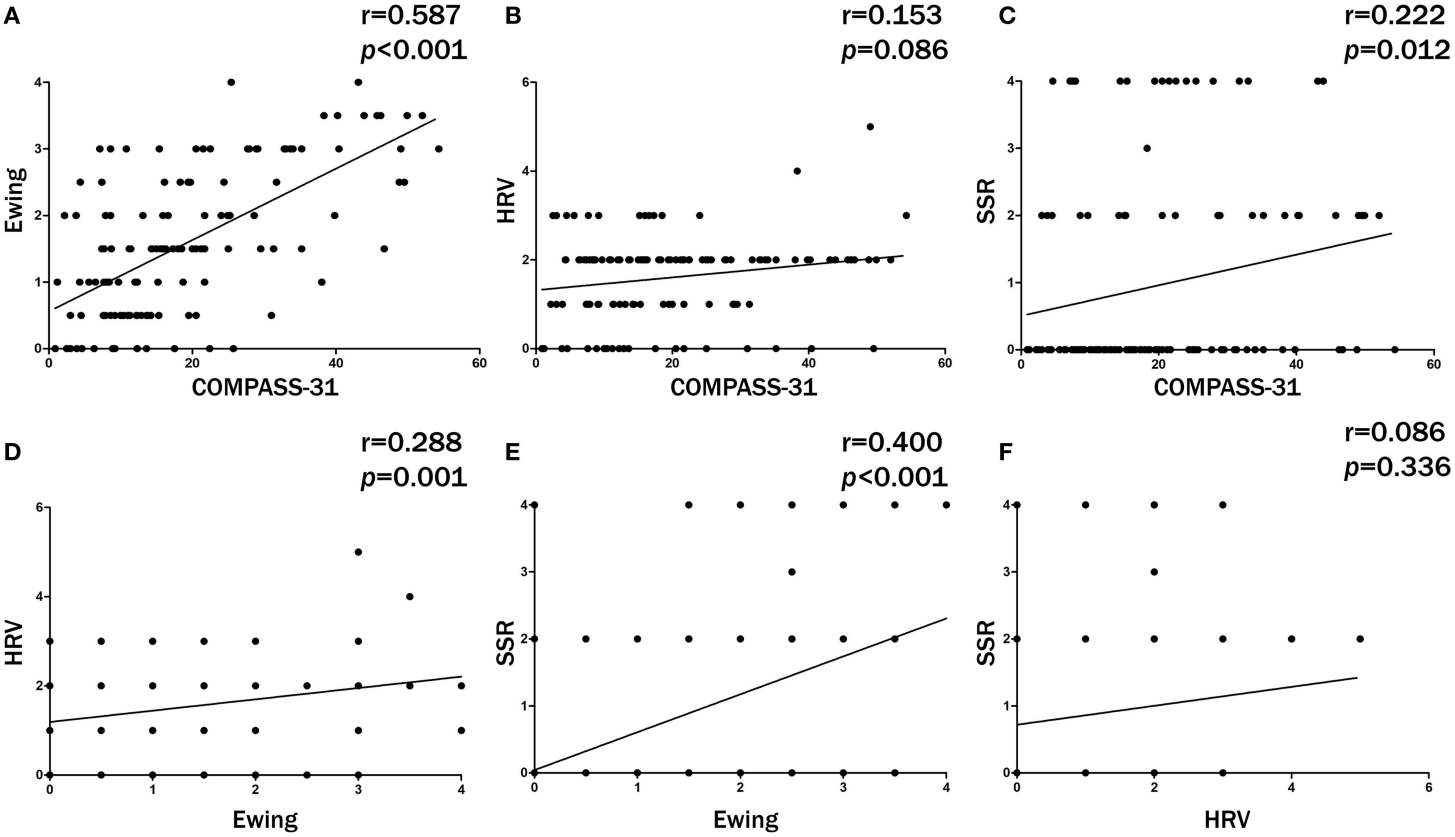
The correlations of diabetic autonomic neuropathy (DAN) severity between any 2 methods. COMPASS-31 and Ewing's test exhibited the best severity correlation. **(A)** Ewing's test versus COMPASS-31. **(B)** HRV versus COMPASS-31. **(C)** SSR versus COMPASS-31. **(D)** HRV versus Ewing's test. **(E)** SSR versus Ewing's test. **(F)** SSR versus HRV.

The agreement of diagnosing DAN between any two methods is shown in Table 3. The 2 × 2 tables are shown on the bottom left corner, and the kappa consistency coefficients are shown on the top right corner. The results indicated that COMPASS-31 and Ewing's test had the best consistency for diagnosing DAN (κ = 0.512, closest to 0.75). The correlations of the severity of DAN scored by each of the methods are shown in Figure 1. The results indicated that COMPASS-31 and Ewing's test exhibited the best correlation (*r* = 0.587) with respect to diagnosing DAN severity.

**Table 3:** The kappa consistency tests of the four methods for diagnosing diabetic autonomic neuropathy (DAN).

	**COMPASS-31**			**Ewing**			**HRV**			**SSR**
COMPASS-31	–			κ = 0.512 *p* < 0.001			κ = 0.152 *p* = 0.050			κ = 0.273 *p* = 0.002
Ewing		DAN(+)	DAN(–)	–			κ = 0.299 *p* < 0.001			κ = 0.354 *p* < 0.001
	DAN(+)	33	18							
	DAN(–)	11	64							
HRV		DAN(+)	DAN(–)		DAN(+)	DAN(–)	–			κ = 0.087 *p* = 0.251
	DAN(+)	32	45	DAN(+)	41	36				
	DAN(–)	12	37	DAN(–)	10	39				
SSR		DAN(+)	DAN(–)		DAN(+)	DAN(–)		DAN(+)	DAN(–)	–
	DAN(+)	22	19	DAN(+)	27	14	DAN(+)	28	13	
	DAN(–)	22	63	DAN(–)	24	61	DAN(–)	49	36	

*The kappa coefficients and p-values are shown on the top right corner. The original cases for the consistency of DAN between any two methods are shown in the 2 × 2 tables on the bottom left corner*.

*COMPASS-31, Composite Autonomic Symptom Score 31; HRV, heart rate variability; SSR, skin sympathetic reaction*.

**Figure 2:**
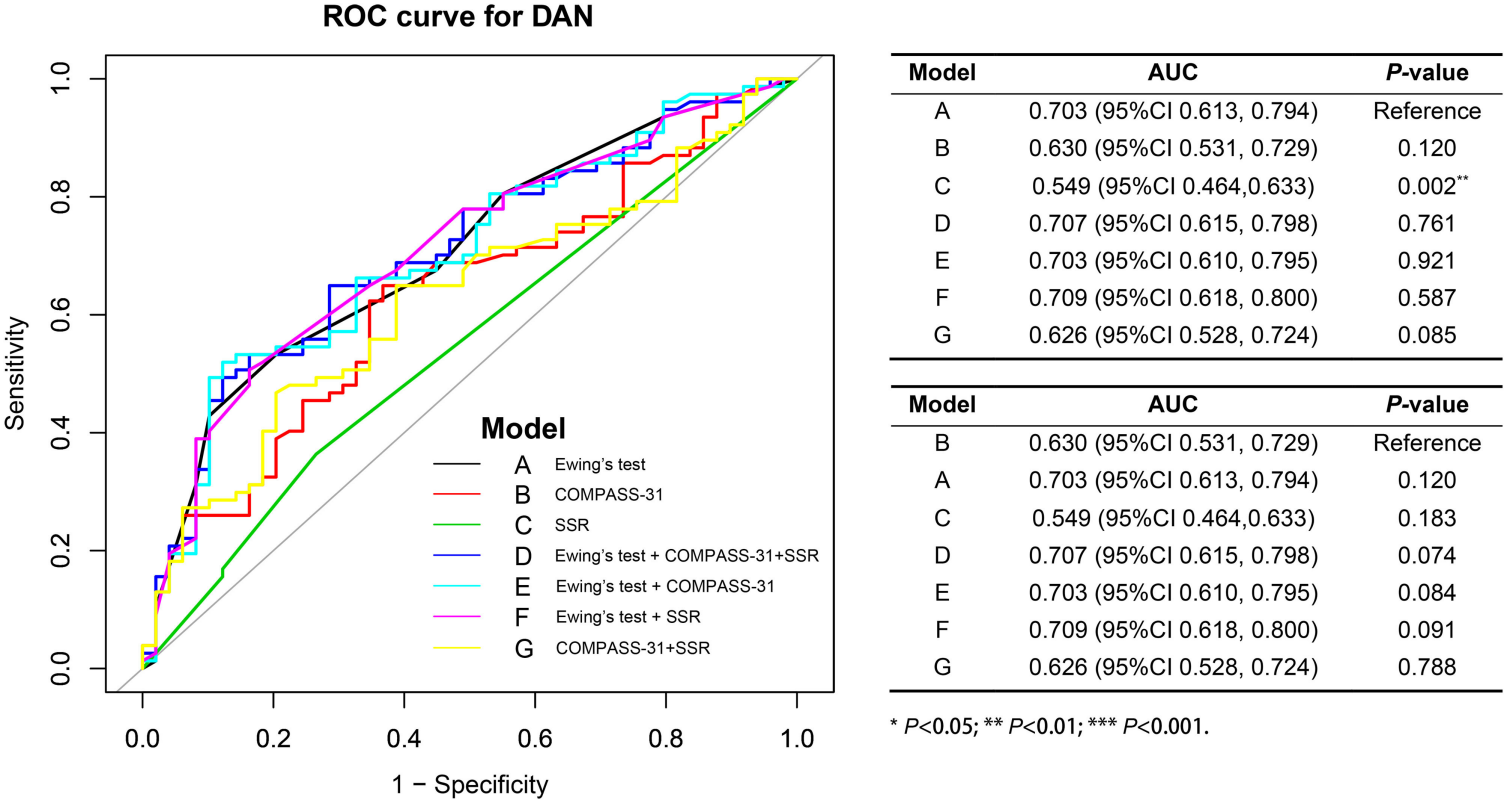
Receiver operating characteristic (ROC) analyses for the diagnosis of diabetic autonomic neuropathy (DAN). Model A (*black*) represents the ROC curve of Ewing's test; model B (*red*) represents the ROC curve of COMPASS-31; model C (*green*) represents the ROC curve of SSR; model D (*deep blue*) represents the ROC curve of Ewing's test combined with COMPASS-31 and SSR; model E (*light blue*) represents the ROC curve of Ewing's test combined with COMPASS-31; model F (*purple*) represents the ROC curve of COMPASS-31 combined with SSR; model G (*yellow*) represents the ROC curve of Ewing's test combined with SSR.

**Figure 3:**
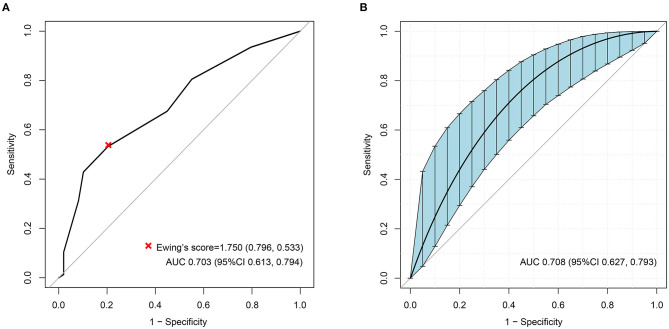
**(A)** Receiver operating characteristic (ROC) curve of Ewing's test to diagnose diabetic autonomic neuropathy (DAN). The optimal cutoff value for classification is indicated by a *multiplication symbol* annotating this threshold value followed by specificity and sensitivity. *AUC*, area under curve. Cutoff value = 1.750, AUC = 0.703, 95% CI = 0.613–0.794, specificity = 79.6%, sensitivity = 53.3%. **(B)** ROC curve of Ewing's test to DAN as calculated by Bootstrap. The *blue shading* denotes the Bootstrap-estimated 95% confidence interval with the AUC. AUC = 0.708, 95% CI = 0.627–0.793.

**Figure 4:**
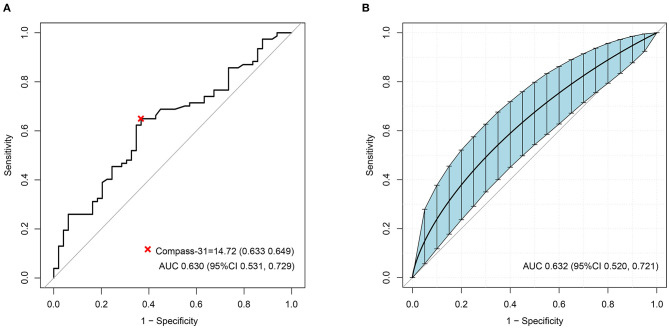
**(A)** Receiver operating characteristic (ROC) curve of COMPASS-31 to diagnose diabetic autonomic neuropathy (DAN). The optimal cutoff value for classification is indicated by a *multiplication symbol* annotating this threshold value followed by specificity and sensitivity. *AUC*, area under curve. Cutoff value = 14.72, AUC = 0.630, 95% CI = 0.531, 0.729, specificity = 63.3%, sensitivity = 64.9%. **(B)** ROC curve of Ewing's test to DAN as calculated by Bootstrap. The *blue shading* denotes the Bootstrap-estimated 95% confidence interval with the AUC. AUC = 0.632, 95% CI = 0.520–0.721.

The ROC analyses of each method by using HRV as the dependent variable are shown in Figure 2. Ewing's test and COMPASS-31 had relatively good diagnostic values [area under the curve (AUC) = 0.703 and 0.630, respectively] which were not inferior to the combined diagnosis. The best cutoff values were 1.75 for Ewing's test (Figure 3A) and 14.72 for COMPASS-31 (Figure 4A). The ROC analyses of Ewing's test and COMPASS-31 calculated by Bootstrap also had good AUC and confidence interval (Figures 3B, 4B).

## Discussion

There are many methods to evaluate autonomic nerve function in clinical practice (17), but the results of the different methods are often inconsistent. Even Ewing's test and HRV results, the two methods recommended by the ADA guidelines for diagnosing DCAN, are often inconsistent in the same patient. Past studies have compared two different methods for assessing autonomic nerve function (18, 19), but only Singh et al. (20) compared the results of COMPASS-31, SSR, Ewing's test, and HRV. The authors divided the patients with diabetes into a definite DCAN, early DCAN, and a no DCAN group using the COMPASS-31 results and then compared the results of the four methods in these three groups. In our study, we also evaluated the autonomic nerve function of diabetic patients with these four methods, but we focused on evaluating the degree of agreement of the four methods in diagnosing DAN.

Because there was not a formal Chinese version and norm of the COMPASS-31, we recruited 84 healthy volunteers as a control group. We found that the COMPASS-31 scores of the control group were not normally distributed and had no correlation with age (data not shown). Furthermore, the average age of the control group was younger than that of the diabetic group. The control group was not large enough and not balanced across age groups. It was the main limitation of this study. In any case, there must be an existing cutoff value. Our cutoff value is slightly lower than that used in a prior study (21.4 vs. 28.7) (20), but is closer to the cutoff value (14.72, seen in Figure 4A) in the ROC analysis. Of course, more precise cutoff values of the COMPASS-31 for diagnosing DAN will require the recruitment of a large number of volunteers in the future.

We used the kappa consistency test to compare the degree of agreement of diagnosing DAN between any two methods. It is generally considered that if the kappa is >0.75, then the agreement between the two methods is good, while a kappa of <0.4 means that agreement is poor; a kappa between 0.4 and 0.75 is considered to indicate moderate agreement. Our results showed that the kappa value of the COMPASS-31 and Ewing's test was the greatest (0.512), indicating that these two tests had the best agreement. All of the other kappa values were <0.4. In addition, in the correlation analysis of DAN severity, the best correlation was found between the COMPASS-31 and Ewing's test (*r* = 0.587). These results indicate that the COMPASS-31 results are consistent with those of Ewing's test, which is recommended by the ADA guidelines. Furthermore, the ROC analyses also showed that Ewing's test and COMPASS-31 individually had good AUC as well as combined for diagnosing DAN (Figure 2). Importantly, the COMPASS-31 is simple and easy to perform in clinical practice, unlike the complicated Ewing's test.

Another advantage of the COMPASS-31 is that it has a continuous range from 0 to 100, which provides a more accurate evaluation of the severity and progression of DAN. Another study has validated that the COMPASS-31 is useful as an initial screening tool for SFPNs (12). In that study, the average COMPASS-31 scores of SFPN(+) and SFPN(–) patients were 38.8 and 19.6, respectively (*n* = 28 and 38, respectively). These scores were both slightly higher than the scores of our DAN(+) and DAN(–) groups (Table 2). This difference might be due to ethnic differences or differences in the methods of grouping. In addition, our translation of the COMPASS-31 may also have impacted the results (21).

HRV is also recommended by the ADA for diagnosing DCAN, but it showed very poor agreement with the other methods in the present study. In Table 2, the patients were divided into a DAN(+) and a DAN(–) group by COMPASS-31. Theoretically, all six HRV items of the DAN(+) group should be lower than those in the DAN(–) group. But in this study, the RMSSD in the DAN(+) group was higher than that in the DAN(–) group, and the other five items showed no significant differences between the two groups. On the contrary, the Ewing and the SSR scores of the DAN(+) group were significantly higher than those of the DAN(–) group. These results also indicate that the agreement between HRV and COMPASS-31 is poor. There were 77 out of 126 diabetic patients (61%) diagnosed as DAN(+) by HRV, and thus the diagnostic rate was much higher than those of the other three methods. We thought that it was because of the poorer discrimination for the severity evaluation of DAN by HRV. We all know that the normal reference value for HRV decreases with age (22). The HRV score has six items. When the HRV is used for diagnosing DAN, a value far below the normal reference value was chosen as a threshold for each item, and so each item is classified as normal or abnormal. Thus, the HRV score ranges from 0 to 6 (seven grades), which is less than the nine grades of the Ewing's test score. In this situation, some diabetic patients with only slight autonomic nervous abnormalities may be over-diagnosed with DAN. In our study, most DAN(+) patients (62/77) diagnosed by HRV had a score of 2 (Figure 1B), which was the lowest threshold for diagnosing DAN. We can speculate that if the six items of HRV were also classified as normal, borderline, and abnormal and scored as 0, 0.5, and 1, respectively, like the Ewing's test, some DAN(+) patients might be scored 1.5 or 1 and would then be categorized as DAN(–). With this method, the diagnosis of DAN with HRV might be improved and the HRV results would be more accurate.

SSR has a similar defect as HRV as the SSR score ranges from 0 to 4 (five grades); SSR exhibited a worse differentiation of DAN than did HRV. Moreover, the disagreement between SSR and Ewing's test and other methods might also be due to anatomical differences of the peripheral autonomic nerves and cardiac autonomic nerves (23, 24). SSR is a measure of peripheral sympathetic function, while three of the four Ewing's test items are a measure of vagus function. However, in the present study, we did not find that DM would tend to involve the peripheral autonomic nerve or the cardiac autonomic nerve first.

There are also some other limitations of the current study that should be considered. Skin biopsy and determination of the intra-epidermal nerve fiber density (IENFD) is the gold standard for the diagnosis of DAN (25), and this was not done. As such, we could not calculate the sensitivity and specificity of each method in the diagnosis of DAN and, thus, not directly evaluate which method is the most accurate. Other methods or questionnaires for the evaluation of autonomic nerve function, such as quantification of nerve fibers in corneal confocal microscopy (NF-CCM) (26), the small fiber neuropathy and symptoms inventory questionnaire (SFN-SIQ), and the small fiber neuropathy screening list (SFNSL) (27, 28), were not evaluated in this study. A comparison of their results with those of COMPASS-31 would be useful. Lastly, creation and validation of a formal Chinese version and normal references of COMPASS-31 should be done to examine the results of this study.

In conclusion, in the present study, the results of COMPASS-31, SSR, Ewing's test, and HRV were compared with respect to the diagnosis of DAN in diabetic patients. Of the four methods, COMPASS-31 and Ewing's test exhibited the best diagnostic agreement and severity correlation, and they had good diagnostic values in the ROC analysis. As COMPASS-31 is a simple, economical, and practical clinical questionnaire, and is much easier to perform than the Ewing's test, it can be used for the diagnosis and follow-up of diabetic patients with DAN.

## Data Availability Statement

The original contributions presented in the study are included in the article/supplementary material, further inquiries can be directed to the corresponding authors.

## Ethics Statement

The studies involving human participants were reviewed and approved by Ethics Committee of Nanfang Hospital (NFEC-2018-115). The patients/participants provided their written informed consent to participate in this study.

## Author Contributions

YP and Y-sL contributed equally to the paper and wrote the first draft. YP, S-yP, and L-lX conceptualized the study design. Y-sL, M-yW, C-nC, C-qL, A-qJ, C-xL, YW, and GT participated in the literature search, data acquisition, performed the data analysis, and statistical analysis. S-yP and L-lX finalized the manuscript. All authors participated in the discussion and approved the final version of the manuscript.

## Conflict of Interest

The authors declare that the research was conducted in the absence of any commercial or financial relationships that could be construed as a potential conflict of interest.
